# Evaluating the efficacy and cost-effectiveness of web-based indicated prevention of major depression: design of a randomised controlled trial

**DOI:** 10.1186/1471-244X-14-25

**Published:** 2014-01-31

**Authors:** Claudia Buntrock, David D Ebert, Dirk Lehr, Pim Cuijpers, Heleen Riper, Filip Smit, Matthias Berking

**Affiliations:** 1 Innovation Incubator, Division Health Training Online, Leuphana University, Lueneburg, Germany; 2 Department of Psychology, Clinical Psychology and Psychotherapy, Philipps University Marburg, Marburg, Germany; 3 GGZ inGeest, Regional Mental Health Service Centre, VU University Medical Centre, Amsterdam, The Netherlands; 4 Department of Clinical Psychology and EMGO Institute for Health and Care Research, VU University, Amsterdam, The Netherlands; 5 Department of Public Mental Health, Netherlands Institute of Mental Health and Addiction, Trimbos Institute, Utrecht, The Netherlands; 6 Department of Epidemiology and Biostatistics, EMGO Institute for Health and Care Research, VU University Medical Center, Amsterdam, The Netherlands

**Keywords:** Prevention, Indicated, Web-based, Subthreshold depression, Randomised controlled trial, Major depression, Cost-effectiveness

## Abstract

**Background:**

Major depressive disorder (MDD) imposes a considerable disease burden on individuals and societies. Web-based interventions have shown to be effective in reducing depressive symptom severity. However, it is not known whether web-based interventions may also be effective in preventing the onset of MDD. The aim of this study is to evaluate the (cost-) effectiveness of an indicated web-based guided self-help intervention (GET.ON Mood Enhancer Prevention) on the onset of MDD.

**Methods/Design:**

A randomised controlled trial (RCT) will be conducted to compare the (cost-) effectiveness of the GET.ON Mood Enhancer Prevention training with a control condition exclusively receiving online-based psychoeducation on depression. Adults with subthreshold depression (N = 406) will be recruited from the general population and randomised to one of the two conditions. The primary outcome is time to onset of MDD within a 12-months follow-up period. MDD will be assessed according to DSM-IV criteria as assessed by the telephone-administered Structured Clinical Interview for DSM-IV (SCID). Time to onset of MDD will be assessed using life charts. Secondary outcomes include changes on various indicators of depressive symptom severity, anxiety and quality of life from baseline to post-treatment, to a 6-month and a 12-month follow up. Additionally, an economic evaluation using a societal perspective will be conducted to examine the intervention’s cost-effectiveness.

**Discussion:**

This is one of the first randomised controlled trials that examines the effect of an indicated guided self-help web-based intervention on the incidence of major depression. If shown to be effective, the intervention will contribute to reducing the disease burden due to MDD in the general population.

**Trial registration:**

German Clinical Trial Registration DRKS00004709.

## Background

Major depressive disorder (MDD) is highly prevalent [1,2] and has an incidence rate that is high relative to the number of prevalent cases [3]. The global point prevalence is estimated at 4.7% with an annual incidence rate of 3% [4]. Moreover, MDD is related to poorer quality of life [5,6], increased mortality [7], and substantial economic costs [8-10]. Currently, MDD ranks as the fourth disorder with the highest disease burden and is projected to be the leading cause of premature mortality and disability in high-income countries by 2030 [11].

The disease burden attributable to MDD might be reduced in two ways. The first approach is to treat existing cases. But despite the availability of effective MDD treatments, such as face-to-face cognitive-behaviour therapy, behavioural activation therapy or problem-solving therapy [12-14] less than half of depressed patients are recognised and treated [15]. Furthermore, it is estimated that approximately only one third of the disease burden caused by MDD could be averted assuming the hypothetical scenario of 100% coverage and full compliance to evidence-based treatments [16,17].

The second approach is reducing the development of new cases, which requires prevention. Preventive interventions might be capable of contributing to a further reduction in disease burden. A recent meta-analysis of 19 randomised controlled trials demonstrated that preventive interventions based on cognitive behaviour or interpersonal therapy were able to reduce the incidence of MDD by 22% [18]. One of these studies also showed the effectiveness of minimal contact cognitive-behavioural therapy for depression, based on the ‘Coping with Depression’ course [19].

Selective prevention aimed at high-risk groups and indicated preventive efforts that target individuals who show already detectable signs of MDD but who do not yet meet the diagnostic criteria for the disorder were particularly effective. Universal prevention aimed at the general population regardless of any risk profile showed only small effects.

Indicated prevention has been suggested to be more “efficient” than selective prevention [20]. “Efficiency” is here defined in terms of “impact”, that is the number of cases that would be prevented if the targeted risk indicator were fully blocked in the population and “effort” reflecting the number needed to be treated to prevent one new case of MDD. From a clinical point of view, indicated prevention is worthwhile for two reasons. First, subthreshold depression is a highly prevalent condition [21] and the burden posed on people affected and the community is considerable [22,23]. Second, subthreshold depression is a risk indicator for MDD, as the incidence rate of MDD is significantly increased in subjects with subthreshold depression compared to those without ranging from .15 in a general population up to .58 in general medical populations and high risk groups [24].

Despite their effectiveness, currently available indicated preventive face-to-face interventions face some serious limitations. These include (a) difficulties delivering interventions to the community *en masse* due to constraints in the workforce and health care resources [25,26], (b) limited availability of evidence-based interventions and clinicians in routine practice, especially in rural areas, and (c) low participation rates even if access to those interventions is at little or no costs [27]. Therefore, new approaches are needed to enhance the impact of indicated preventive interventions.

Using the Internet to provide (guided) self-help interventions may help to overcome some of the limitations of traditional preventive services. Web-based guided self-help strategies have several advantages over face-to-face approaches. These include: (a) interventions are more easily accessible at any time and place, (b) anonymity is assured when patients want to avoid stigmatisation, (c) a greater potential for the integration of acquired skills in daily life due to an emphasis on the participants’ active role in (guided) self-help interventions [28] (d) participants can work at their own pace and go through materials as often as they want, and (e) elimination of travel time and costs for both participants and clinicians. Finally (f), web-based interventions are easily scalable implying that only a small increase in therapeutic resources is required for reaching a greater proportion of the eligible population using these interventions. Thus, marginal costs per additional user are low due to an economies of scale effect.

Web-based interventions have shown to be well accepted by participants [29,30] and to be effective in the acute treatment of MDD [12] as well as in reducing depressive symptoms both in adults and adolescents [31,32]. However, although several web-based interventions are labelled as preventive interventions, i.e. ‘Colour your life’ [33,34], to the best of our knowledge, no study has yet investigated the (cost-) effectiveness of an indicated guided self-help web-based preventive intervention on the onset of diagnosed major depressive disorders.

### Objective and research questions

The aim of this study is to evaluate whether a newly developed indicated guided self-help web-based intervention (GET.ON Mood Enhancer Prevention) is effective in preventing the onset of major depressive disorder when compared to an online psychoeducation-only control over a 12-months follow-up period. It is expected that depressive symptomatology will be reduced to a greater extend in the intervention group than in the control condition. It is hypothesised that GET.ON Mood Enhancer Prevention is superior in terms of cost-effectiveness, and QALY health gains compared to the psychoeducation-only control.

## Methods/Design

### Design

**Figure 1:**
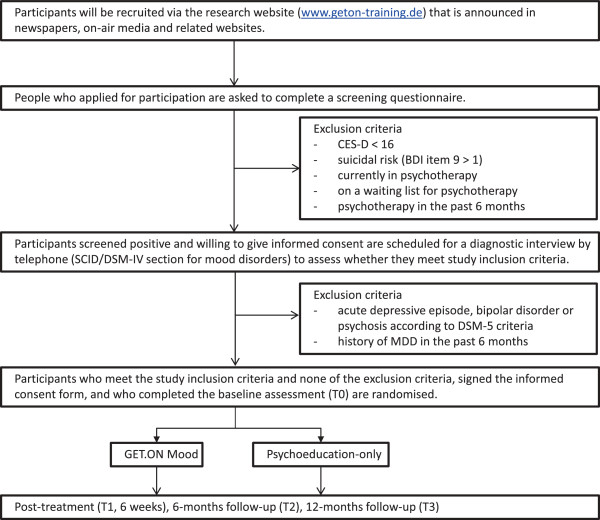
Overview of study procedure.

A two-armed randomised controlled trial (RCT) will be conducted to compare GET.ON Mood Enhancer Prevention with a psychoeducation-only control condition. Measurements will be taken at baseline, post-treatment (6 weeks), 6, and 12 months follow-up (after randomisation). Telephone-administered Semi-Structured Diagnostic Interviews (SCID) will be conducted at baseline, 6- and 12-months follow-up with the SCID/DSM-IV section for mood disorders (see Figure 1 for a detailed overview of the study design). The study is approved by the Medical Ethics Committee of the Philipps University Marburg (No. 2012-35 K).

### Inclusion and exclusion criteria

We will include adults (a) age 18 and above who (b) suffer from subthreshold depression (Centre for Epidemiological Studies Depression Scale (CES-D) ≥ 16) but do not meet DSM-IV criteria for a major depressive episode, (c) have Internet access, and (d) are willing to give informed consent. We will exclude subjects who (a) meet DSM-IV criteria for (a) current major depressive episode, (b) a bipolar disorder, or (c) a psychotic disorder. Additional exclusion criteria are: (d) a history of a major depressive disorder in the past six months (based on Kupfer [35]), (e) currently receiving psychotherapy for any kind of mental health problems, (f) being on a waiting list for psychotherapy, (g) receiving psychotherapy in the past six months, and (h) showing a notable suicidal risk, as indicated through a score greater 1 in the Beck Depression Inventory (BDI) Item 9 (“I feel I would be better off dead”).

### Recruitment

Participants will be recruited from March 2013 to February 2014 via the GET.ON research website [36] that is announced in newspapers, on-air media and related websites. The research website provides information about the GET.ON Mood Enhancer Prevention training and details about the study. Additionally, a major health insurance company will support the recruitment by placing the information leaflet on its website. Individuals interested in participating in the study can apply online on the GET.ON research website by providing the research team with their e-mail address or by sending directly an e-mail to the research team. They do not need to be referred by their GP or other mental health care specialist.

### Assessment of eligibility and randomisation

People who apply online for study participation receive an information letter via e-mail with detailed information about the study procedures. They will be informed that they can withdraw from the intervention and/or study at any time without any negative consequences. Applicants who still want to participate in the study are asked to complete online screening questionnaires including information about the severity of their depressive symptoms (CES-D ≥ 16), whether they are currently receiving any kind of treatment for any mental health disease, whether they are on a waiting list for such a treatment, whether they received such a treatment in the past six months, and whether they have a high suicidal risk (BDI Item 9 > 1). Subjects screened positive and who are willing to give informed consent are scheduled for a semi-structured clinical interview (SCID) conducted by telephone [37,38]. Participants meeting all of the inclusion and none of the exclusion criteria who have completed the baseline assessment and returned the informed consent form via post or e-mail will enter the study and will be randomly allocated to study conditions. Randomisation will take place at an individual level. Block randomisation will be used to ensure equity of ample sizes across study conditions. Random blocks will consist of two allocations each. The allocation will be done by an independent researcher not otherwise involved in the study using an automated computer-generated random numbers table.

### Blinding

The research staff conducting the semi-structured clinical interviews at 6- and 12-months follow-up will be blinded to the condition the participants are assigned to. These include: (a) an explanation to the participants why it is important not to inform the interviewer about the condition they were assigned to; (b) a written reminder for the interviewer in the interview manual to ask the participant not to inform him/her about the randomisation status; (c) written and verbal reminders to the patient before each interview; and (d) a documentation after each assessment of whether or not the interviewer is still blind to treatment condition. With regard to the latter, the interviewer will be asked to guess each participant’s randomisation status and these guesses will be compared with the actual status. Cohen’s kappa will be computed to clarify whether hit rates differ from what can be expected from chance. In case of evidence for blinding break down, the interviewer will be changed to the second outcome interview.

### Intervention

#### Get.ON mood enhancer prevention

The GET.ON Mood Enhancer Prevention training consists of six lessons. Participants are advised to do two lessons a week but at least one. Consequently, the training lasts 3 to 6 weeks. However, participants are not excluded from the intervention if they do not manage to complete one lesson a week. Lessons consist of text, exercises, and testimonials. Each lesson includes interactive elements such as audio and video clips. Audio sequences introduce relaxation exercises, whereas video clips are used to explain theoretical frameworks, such as the concept of behavioural activation, in a user-friendly way. A strong focus lies on transfer tasks (homework assignments) to integrate newly acquired strategies and techniques into daily life. As an optional component, participants can choose to receive a set of about 42 standardised text-messages supporting them to integrate the learned techniques into their everyday life. An example of such a text-message would be “Everyone has his own strategies to vanquish the inner temptation. What helps you?” In the beginning of each subsequent lesson, participants are invited to reflect on their experiences with the newly acquired skills. The contents are adaptively tailored to the specific needs of the individual participant by continuously asking participants to respond by choosing among various response options. Subsequent content is then tailored to the participant’s response. For example, participants are asked whether to work on an elective module or not and if so they can choose which module they want to work on.

GET.ON Mood Enhancer Prevention is based on elements from behaviour therapy (BT) [39] and problem-solving therapy (PST) [40]. These therapeutic elements are often found in psychological treatments for subthreshold depression [41]. Interventions using BT and the combination of BT and PST have been shown to effectively prevent the onset of major depressive disorder (i.e. [42,43]). In BT, a strong focus rests on daily pleasurable activity scheduling that is integrated in each lesson (Additional file 1: Screenshots of the GET.ON Mood Enhancer Prevention intervention page 3). The PST elements implemented in GET.ON Mood Enhancer Prevention have been used in various web-based interventions, such as the Dutch web-based “Alles onder Controle” course, which has been shown to be effective in reducing depressive symptomatology across several randomised controlled trials [44,45]. In the current study, PST consists of three steps. First, participants make a list of things that matter most to them in their lives. Second, participants list all their problems and worries and divide them into ‘manageable’ and ‘unmanageable’. Finally, they are invited to think about activities on how to solve the manageable problems. The main focus in problem-solving therapy is to tackle those problems that are manageable by means of a six-step procedure: (1) defining the problem, (2) defining the target state, (3) brainstorming about possible solutions and choosing the best one, (4) making a plan how to implement this solution, (5) actually putting the solution into practice, and (6) evaluating the outcome. Finally, participants make a plan for the future on how they are going to accomplish their goals and those things that are most important to them in their lives (Additional file 1 page 2). In addition to the BT and PST elements, in the last three lessons participants are offered three elective modules targeting sleep hygiene, relaxation techniques, and dealing with worrying thoughts, respectively.

During the training, participants are supported by an online-trainer. The total time a trainer spends on a participant is approximately two hours. Trained and supervised graduate students and health care professionals will provide guidance. Participants will communicate with their trainer trough the internal messaging function of the system on which GET.ON Mood Enhancer Prevention is implemented. The guidance provided by the trainers focus on supporting participants to work through the exercises.

#### Psychoeducational-only condition

The psychoeducational intervention is also web-based and it is implemented on the same platform as GET.ON Mood Enhancer Prevention. Psychoeducational interventions have been shown to be effective in reducing depressive symptoms [46]. In the current study, the psychoeducational intervention is based on the German S3-Guideline/National Disease Management Guideline Unipolar Depression [47]. It informs participants about the nature and evidence-based treatments of depression including information about symptoms and sources of help. They can go through the material as often as they want to. In this study, the psychoeducational intervention does neither require participants to do explicit homework assignments nor is any support by a trainer or other mental health care specialist offered to participants.

### Sample size calculation

We assume an absolute risk reduction of at least 10% for the incidence of major depressive disorder (MDD) between intervention and control group as clinically relevant. Based on previous studies evaluating interventions directed at the prevention of MDD, we expect a mean incidence of MDD in the control group of 25% within the 12-months follow-up period [48-50]. Based on a power of 80%, an alpha of 0.05, a relative risk reduction of 40%, and an attrition rate of 20%, we will need 406 participants to demonstrate an absolute risk reduction of 10% between the groups using log rank survival analyses (calculated using PASS 12).

### Outcome measurements

For an overview of assessment at baseline, post-treatment, 6-and 12-month follow-up see Table 1.

**Table 1:** Overview of outcome measurements

		**Time of measurement**			
**Instrument**	**Aim**	**T0 (Baseline)**	**T1 (Post-test, 6 weeks)**	**T2 (6-month follow-up)**	**T3 (12-month follow-up)**
**Other questions**	Socio-demographics	x			
**SCID** (DSM-IV section for mood disorders)	Diagnostic interview	x		x	x
**CES-D**	Depressive symptom severity	x	x	x	x
**EuroQol**	Quality of life	x	x	x	x
**SF-12**	Subjective functioning/Quality of life	x	x	x	x
**TiC-P**	Health care service utilisation and productivity losses	x		x	x
**HADS-A**	Anxiety symptoms	x	x	x	x
**SPSI-R**	Problem-solving skills	x	x	x	x
**BADS-SF**	Behavioural activation	x	x	x	x
**Pearlin Mastery Scale**	Internal locus of control	x	x	x	x
**PSWQ** (Ultra-brief)	Worrying thoughts	x	x	x	x
**ISI**	Insomnia Severity	x	x	x	x
**CEQ**	Patient expectancy/treatment credibility	x	x		
**ATSPPH-SF**	Attitudes toward seeking professional psychological help	x	x	x	x
**Course evaluation**	Participants’ satisfaction with the GET.ON Mood Enhancer Prevention training		x		
**INEP**	Side-effects of psychotherapy		x	x	

#### Primary outcome

The primary outcome is time to onset of MDD within a 12-months follow-up period. Major depressive disorder will be assessed according to DSM-IV criteria as assessed by the telephone-administered Structured Clinical Interview for DSM-IV (SCID) at 6 and 12 months [37,38]. The inter-rater agreement of the Axis I disorders is moderate to excellent [51]. The agreement between face-to-face and telephone SCID interviews as indicated by the kappa coefficient is considered to be excellent [52]. Time to onset of MDD will be assessed using life charts. Life events are recalled by using a calendar method after which the presence of depressive symptoms at each month during the follow-up period is determined. SCIDs will be conducted by trained psychologists who are blind to treatment condition. The interviews will be recorded to examine inter-rater reliability. Disagreement shall be solved by discussion and the agreed rating will be used for analysis. If this is not possible, the assessment will be rated by an experienced psychotherapist (gold standard) and this rating will be used for analysis.

### Secondary outcomes

Self-report data will be collected using a secured online-based assessment system (AES, 256-bit encrypted).

#### Depressive symptomatology

The depressive symptom level will be assessed with the German version of the Center for Epidemiological Studies Depression Scale (CES-D) [53]. The CES-D is a self-report scale and consists of 20 items, each scored 0–3, covering four domains: depressive affect, somatic complaints/activity inhibition, positive affect, and interpersonal difficulties. The total score ranges from 0–60, with a higher score indicating more severe depressive symptoms. A cut-off of 16 is usually regarded as indicating clinically relevant depressive symptom severity. The reliability of the CES-D has been shown to be excellent (internal consistency of Cronbach’s α = .89) [53].

#### Quality of life

Health-related quality of life will be assessed with two multidimensional generic measures, i.e., the EuroQol [54] and the SF-12v1 Health Survey [55]. The EuroQol entails the EQ-5D and a visual analogue scale. The EQ-5D consists of five items covering five dimensions (mobility, self-care, usual activities, pain/discomfort, and anxiety/depression), each of which is rated as causing ‘no problems’, ‘some problems’, or ‘extreme problems’. The SF-12v1 has 12 items covering eight health domains (physical functioning, role functioning (physical and emotional), bodily pain, general health, vitality, social functioning, and mental health). The SF-12 generates two summary scores, the physical and mental health summary scores, respectively.

#### Anxiety

Anxiety will be measured with the German version of the anxiety subscale of the Hospital Anxiety and Depression Scale (HADS-A) [56,57]. The anxiety subscale consists of seven questions and each is scored from 0-3 meaning that the total scores ranges from 0-21 where a score between 0-7 indicates no anxiety, between 8 and 10 possible anxiety, and above 11 or 12 a clinical anxiety disorder. Psychometric properties are well established (Cronbach’s α ranging from .63-.93) [58].

#### Problem-solving skills

Problem-solving ability (i.e., generalised appraisal, beliefs, expectancies, and emotional responses) will be measured with two subscales of the Social Problem-Solving Inventory-Revised (SPSI-R). The positive problem orientation (PPO) subscale will represent a constructive dimension whereas the negative problem orientation (NPO) subscale is viewed as a dysfunctional dimension. Both subscales have displayed strong psychometric properties in former studies (Cronbach’s α = .76; .83) [59].

#### Behavioural activation

Participants’ activation towards goals/values and pleasant activities and avoidance behaviours will be measured with the BADS-Short Form (BADS-SF) [60]. The BADS-SF entails 9 items comprising two subscales (activation and avoidance). The items are rated on a 7-point Likert-type scale. Higher scores indicate that the individual scores high on the area of interest. The BADS-SF shows good psychometric properties (Cronbach’s α = .82) [60].

#### Mastery (internal locus of control)

Internal locus of control will be measured with the Pearlin Mastery Scale [61]. The Pearlin Mastery Scale consists of 7 items and each is rated on a 4-point Likert scale. The higher the score, the more the individual perceives having control over situations (internal mastery). A lower score points to external mastery meaning that the individual generally has the feeling that things are out of his or her control. The psychometric properties of this scale are well established [61].

#### Worrying

Worrying will be assessed with the ultra-brief version of the Penn State Worry Questionnaire (PSWQ) [62]. The ultra-brief version consists of 3 items stemming from the standard version, with each item being rated on a 7-point scale. The total score range from 0-18 with higher scores indicating more worry. The ultra-brief version shows similar psychometric properties compared to the standard version (Cronbach’s α = .85) [62].

#### Insomnia severity

Insomnia severity will be measured because evidence shows that treating sleep problems can ease depressive symptoms. Insomnia severity will be assessed with the Insomnia Severity Index (ISI) [63]. The ISI measures the nature, severity, and impact of insomnia. It consists of 7 items; each is rated on a 5-point Likert scale resulting in a total score ranging from 0 to 28. Higher scores indicate more severe insomnia. The ISI is a valid and reliable instrument to detect cases of insomnia in a population-based sample. The internal consistency is excellent (Cronbach’ α = .90) [64].

#### Treatment credibility/patient expectancy

Training credibility and participants’ expectancy for improvement will be measured with the credibility and expectation questionnaire (CEQ). The CEQ consists of 6 items, which are rated on a 9- or sometimes 10-point Likert scale. The psychometric properties of the instrument are well established (Cronbach’s α = .86) [65].

#### Attitudes toward seeking professional psychological help

The influence of attitudes on mental health care service utilisation will be measured with the Attitudes Toward Seeking Professional Psychological Help Scale-SF (ATSPPH-SF) [66]. The ATSPPH-SF consists of 10 items that are rated on a 4-point Likert scale yielding a total score ranging from 0-30. High scores indicate positive treatment attitudes. The instrument showed good psychometric properties in a previous study. The internal consistency ranges from .82 to .84 [67].

#### Course evaluation

User satisfaction will be measured with a self-designed questionnaire that is based on the “Satisfaction with Psychotherapy” Questionnaire (ZUF-8, [68]), the German version of the Client Satisfaction Questionnaire (CSQ-8, [69]). This self-report measure consists of 8 items measuring the global client satisfaction with the web-based training. Previous research indicated a high internal consistency (Cronbach’s α = .91) [70].

#### Side-effects of psychotherapy

Side-effects of psychotherapy will be measured with the side-effects of psychotherapy inventory (INEP) [71]. The INEP consists of 15 items assessing any changes participants experienced after the completing of the web-based training in their social and/or work environment that they directly relate to their participation in the web-based training.

#### Key economic outcomes

##### Clinical endpoints

In the cost-effectiveness analyses, the main outcome will be depression-free years gained. Depression-free years will be assessed by calculating the difference in follow-up lengths and the duration of any major depressive episode (i.e. period of time in weeks that a person met DSM-IV criteria). In the cost-utility analysis, quality-adjusted life years (QALYs) will be the clinical endpoint. QALYs will be obtained from the EQ-5D (EuroQol) and SF-6D (SF-12v1). The EuroQol will be used because it is a widely applied quality of life instrument and its reliability and validity is well established [54]. Theoretically, the EQ-5D generates 243 different health states. Index scores for each of these health states are available for various countries with “perfect health” and “death” being assigned values of 1 and 0, respectively. In this study, the index scores derived from a large general population sample in the UK will be used [72]. For the sensitivity analysis, the German index scores [73] will be applied. The EQ-5D, however, might suffer from a ceiling effect meaning that a large number of respondents suffering from mild manifestations of depressive symptoms report no problems. Hence, the SF-6D will also be used because it might be the more appropriate measure for milder conditions, i.e., subthreshold depression [74]. The SF-6D contains 6 dimensions (each with between 2 and 5 levels) and includes 7 items of the SF-12. The SF-6D generates 7500 different health states. Utility values will be derived using Brazier’s algorithm [75,76].

##### Costs

Direct medical (i.e. health care service utilisation) and direct non-medical costs (i.e. all costs not directly related to medical services such as transportation) are measured with the TiC-P, which is a self-report questionnaire [77]. Indirect non-medical cost stemming from production losses due to absenteeism and presenteeism will be assessed with specific modules of the TiC-P. A catalogue of unit costs (i.e. inpatient, outpatient and rehabilitative services) and evaluation standards of the Association of Statutory Health Insurance Physicians [78] will be used to calculate the total care costs on an individual basis [79].

### Statistical analyses

#### Clinical efficacy

The study will be conducted in agreement with the CONSORT statement. Differences in the time to onset of MDD will be analysed in a proportional log rank survival analysis over a follow-up period of 12 months after baseline. Time to onset will be the dependent variable in the survival analyses. Analyses will be done based on the intention-to-treat (ITT) principle. In addition, per-protocol analyses will be performed. For participants who are lost from the trial, available measurements will be used and then censored at the time of their last observation. Participants who miss the assessment at 6-months follow-up, but are then assessed at 12-months follow-up, will be asked about their current and past symptoms according to SCID diagnostic criteria since the diagnostic interview at baseline. This will enable us to assess the time to onset of a depressive episode and thus to censoring. One-sided tests will be used for testing unidirectional and two-sided tests for testing bidirectional hypotheses. For all analyses statistical significance will be set at p < .05. We will calculate the number needed-to-be-treated (NNT) with GET.ON Mood Enhancer Prevention to prevent one case of MDD as compared to the control group to estimate the clinical effect size.

In secondary analyses, hierarchical linear modelling will be used to assess differences in secondary outcomes, such as depressive symptom severity, anxiety, and quality of life. We will also explore the proportion of major depressive episodes in the intervention and control group by follow-up. For all mixed-model analyses, Cohen’s d will be calculated by standardising the differences between baseline and follow-up scores by the pooled standard deviation of baseline scores.

#### Economic analyses

It will be checked whether baseline differences exist between the intervention and control group. If necessary, statistical techniques will be used to correct for baseline differences [80]. In the cost-effectiveness analyses, the incremental cost-effectiveness ratio (ICER) will be stated as costs per depression-free years gained, whereas the ICER in the cost-utility analyses will represent the costs per quality-adjusted life year (QALY) gained. Bootstrapping will be used to test the robustness of the ICERs and quantify the uncertainty around the ratios that will be graphically represented on a cost-effectiveness plane. The bootstrapped ICERs will also be shown in a cost-effective acceptability curve disclosing the probability that GET.ON Mood Enhancer Prevention is cost-effective for a wide range of willingness-to-pay ceilings [81]. To test the robustness of the base-case findings, a multi-way sensitivity analysis will be done. An incremental net benefit regression analysis will be performed to ascertain which sub-groups benefit more from the intervention in terms of superior cost-effectiveness.

## Discussion

Major depressive disorder is a highly prevalent disorder associated with a considerable loss of quality of life, increased mortality rates, and formidable economic costs. Due to limited accessibility and efficacy, treating existing cases only contributes to a partial reduction of disease burden. Thus, interventions preventing the onset of MDD should be used to complement treatment-focused interventions and further reduce the burden of this debilitating disorder. Available face-to-face preventive interventions face, however, limitations. Novel approaches are needed that go beyond the limits of traditional services. The Internet may attract people who do not participate in face-to-face interventions. Moreover, it potentially provides the opportunity to offer preventive interventions to the community *en masse*. To our knowledge, this is one of the first randomised controlled trials that examines the (cost-) effectiveness of a web-based intervention on the onset of major depression in subjects with subthreshold depression.

Limitations of this study include the following. Attrition is a common problem in web-based interventions [82]. However, providing guidance has been shown to reduce attrition rates [12] and the intensity of support offered in this study is considered to keep drop-out to a minimum. In addition, the psychometric properties of most of the secondary outcome measures used in this trial have not yet been tested in an online environment.

There are several strengths to this study. First, by conducting this trial a significant contribution to the literature is made as to the best of our knowledge no other study has yet been undertaken that investigates the effect on an indicated guided self-help web-based intervention on the incidence of MDD. Second, Semi-Structured Diagnostic Interviews (SCID) will be conducted two times within the 12-month follow-up period to assess the time to onset of depressive episodes. This frequency of assessments allows for a reasonable temporal precision of onset of depressive episodes according to DSM-IV criteria. Third, an economic evaluation will be performed alongside the randomised controlled trial. If shown to be effective, this web-based preventive intervention could be easily disseminated. As a low-threshold intervention, it would be better accepted among the target group. If shown to be cost-effective, GET.ON Mood Enhancer Prevention will be a valuable tool to efficiently reduce the disease burden attributable to MDD at population level.

## Competing interests

Professor Berking is minority shareholder of Minddistrict GmbH, which will provide the platform for the web-based intervention.

## Authors’ contributions

MB obtained funding for this study. MB, CB, JR, LB, DE, and DL contributed to the development of the GET.ON Mood Enhancer Prevention training. All authors contributed to the study design. FS contributed to the design of the economic evaluation study. CB drafted the manuscript. CB, DE, PC, FS, and MB contributed to the further writing of the manuscript. All authors read and approved the final manuscript.

## Pre-publication history

The pre-publication history for this paper can be accessed here:

http://www.biomedcentral.com/1471-244X/14/25/prepub

## Supplementary Material

Additional file 1 Screenshots of the GET.ON Mood Enhancer Prevention intervention. Click here for file
