# Supplementary material for: Evaluating the efficacy and cost-effectiveness of web-based indicated prevention of major depression: design of a randomised controlled trial
Source: BMC Psychiatry. 2014 Jan 31;14:25. doi: 10.1186/1471-244X-14-25 (PMC3914724; doi:10.1186/1471-244X-14-25)
Supplement: Additional file 1 — Screenshots of the GET.ON Mood Enhancer Prevention intervention. [file 1471-244X-14-25-S1.pdf]

Erika Mustermann | Benutzerprofil | Abmelden

GET.ON
Gesundheits
Training.Online

Start

- Aufgaben (1)
- Nachrichten
- Kontaktpersonen

Programm

- GET.ON Stimmung

Tagebuch

- Depression

AUFGABE (1)

Aufgabe verfügbar
Aufgabe "GET.ON Stimmung Lektion 1" verfügbar in "GET.ON Stimmung".

KONTAKTPERSONEN

Claudia Buntrock

NEUE MITTEILUNGEN (0)

Sie haben keine neuen Mitteilungen.

Alle Ihre Mitteilungen ansehen ▶

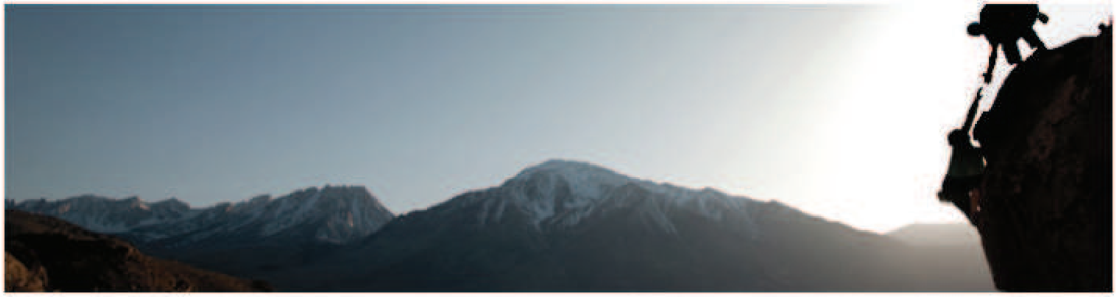

3. Der 6-Schritte-Plan

Der 6-Schritte-Plan aus dieser Lektion soll Sie dabei unterstützen, die Probleme zu bearbeiten, die Sie selbst beeinflussen können. Der Plan kann Sie motivieren und Ihnen Handlungsalternativen aufzeigen, wenn Sie nicht unmittelbar eine Lösung für ein Problem haben.

Wie sehen die 6 Schritte dieses Plans aus?

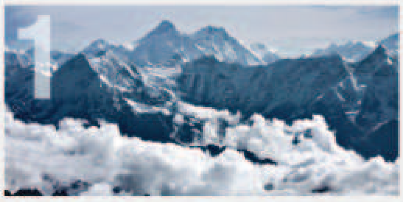

SCHRITT 1:
WAS IST MEIN PROBLEM?

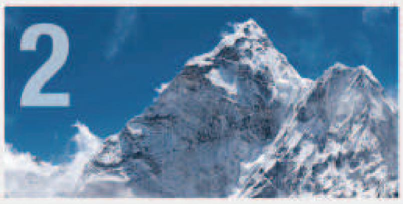

SCHRITT 2:
WAS IST MEIN ZIEL?

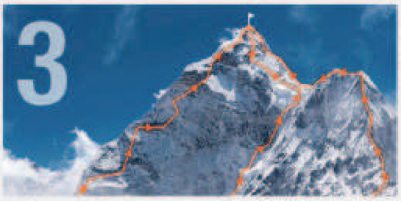

SCHRITT 3:
LÖSUNGSMÖGLICHKEITEN FINDEN

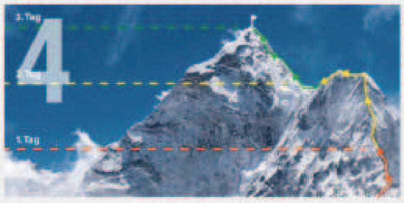

SCHRITT 4:
DIE (KLEINEN) SCHRITTE ZUR PROBLEM-LÖSUNG FESTLEGEN

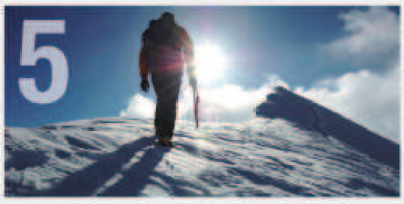

SCHRITT 5:
DIE LÖSUNGSMÖGLICHKEIT AUSPROBIEREN

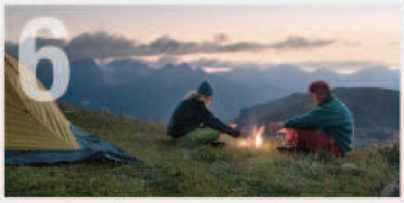

SCHRITT 6:
BILANZ ZIEHEN

Im Folgenden haben Sie die Möglichkeit, Ihren ersten 6-Schritte-Plan zu bearbeiten.

Klicken Sie auf "Weiter", um fortzufahren.

Vorherige: 2. Probleme und depressive Stimmung

Weiter: 4. Mein 6-Schritte-Plan

Planen Sie Ihre Aktivitäten

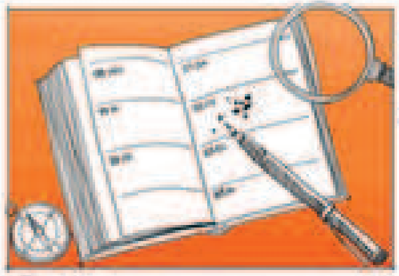

An dieser Stelle laden wir Sie wieder dazu ein, Aktivitäten zu planen, um die wichtigen Dinge in Ihrem Leben weiter zu stärken und Ihre Stimmung weiter zu verbessern. Schauen Sie sich für Ihre Aktivitätenplanung ruhig noch einmal Ihre persönliche Sammlung von Aktivitäten an.

▶ Meine wichtigen Dinge und wie ich diese stärken möchte

▶ Meine positiven Aktivitäten

Wochentag	Aktivität	Mögliches Hindernis	Wie ich das überwinde
-			

✚ Hinzufügen

○ Nicole ○ Jürgen

Nachdem Sie nun Ihre Aktivitäten geplant haben, haben Sie nun die Möglichkeit, noch einmal über Ihre Lernerfahrungen nachzudenken und im Überblick zu sehen, was Sie in der nächsten Lektion erwarten wird.

Klicken Sie auf "Weiter", um diese Lektion zu beenden.

Vorherige: 5. Unlösbare Probleme akzeptieren

Weiter: 7. Zusammenfassung
